# Developing a practice guideline for the occupational health services by using a community of practice approach: a process evaluation of the development process

**DOI:** 10.1186/s12889-016-4010-0

**Published:** 2017-01-18

**Authors:** Lydia Kwak, Charlotte Wåhlin, Kjerstin Stigmar, Irene Jensen

**Affiliations:** 1 Unit of Intervention and Implementation Research for worker health, Institute for Environmental Medicine, Karolinska Institutet, Box 210, 171 77 Stockholm, Sweden; 2 Occupational and Environmental Medicine Center, University Hospital, Region Östergötland, and Department of Clinical and Experimental Medicine, Linköping University, Linköping, Sweden; 3 Physiotherapy, Department of Health Sciences, Lund University, Lund, Sweden; 4 Epidemiology and Register Centre South, Skåne University Hospital, Lund, Sweden

**Keywords:** Practice guidelines, Evaluation, Process measures

## Abstract

**Background:**

One way to facilitate the translation of research into the occupational health service practice is through clinical practice guidelines. To increase the implementability of guidelines it is important to include the end-users in the development, for example by a community of practice approach. This paper describes the development of an occupational health practice guideline aimed at the management of non-specific low back pain (LBP) by using a community of practice approach. The paper also includes a process evaluation of the development providing insight into the feasibility of the process.

**Methods:**

A multidisciplinary community of practice group (*n* = 16) consisting of occupational nurses, occupational physicians, ergonomists/physical therapists, health and safety engineers, health educators, psychologists and researchers from different types of occupational health services and geographical regions within Sweden met eleven times (June 2012–December 2013) to develop the practice guideline following recommendations of guideline development handbooks. Process-outcomes recruitment, reach, context, satisfaction, feasibility and fidelity were assessed by questionnaire, observations and administrative data.

**Results:**

Group members attended on average 7.5 out of 11 meetings. Half experienced support from their workplace for their involvement. Feasibility was rated as good, except for time-scheduling. Most group members were satisfied with the structure of the process (e.g. presentations, multidisciplinary group). Fidelity was rated as fairly high.

**Conclusions:**

The described development process is a feasible process for guideline development. For future guideline development expectations of the work involved should be more clearly communicated, as well as the purpose and tasks of the CoP-group. Moreover, possibilities to improve support from managers and colleagues should be explored. This paper has important implications for future guideline development; it provides valuable information on how practitioners can be included in the development process, with the aim of increasing the implementability of the developed guidelines.

## Background

Non-specific low back pain (LBP) is a major cause of work disability, [1] productivity loss [2] and sick leave [3]. Patients with LBP are often seen in primary health care, but due to the high prevalence of LBP among people of working age, it is a commonly encountered problem within the Occupational Health Services (OHS) [4]. The OHS main assignment is to support employers and employees with work related health issues [5]. This includes a diversity of tasks such as counselling workers with non-specific LBP and assisting them to continue working or to return to work after sick leave [6]. The role of the OHS is unique, as they have the opportunity to assess and intervene on multiple risk factors, including those related to work, [7] this in contrast to, for example, the primary health care.

There is a variation in services provided by the OHS, this despite general recognition of the importance of evidence-based practice [8]. To improve the quality of care provided, there is a need to facilitate the translation of research into the OHS practice. In 2011, the Swedish Research Council for Health, Working Life and Welfare granted us a six year programme, with the aim of enhancing the evidence-base of the OHS in Sweden. Inspired by the Netherlands Society of Evidence-based Medicine’s practice guidelines in occupational health (OH) [8] one of the approaches within the programme is to develop practice guidelines to assist OHS professionals’ decisions about appropriate work related assessments and interventions.

The mere existence of evidence-based guidelines does however not guarantee their usage, as many studies have demonstrated lack of compliance [9]. Several factors influence guideline adherence, including guidelines’ scientific evidence, relevance and applicability, and whether they contain implementation information [10]. The field of OHS research is growing, however research evidence in it is still limited [11–13]. With regards to the relevance and applicability of occupational health (OH) guidelines, contextual information explicit to the OHS is often lacking. OH guidelines for the management of LBP for example lack recommendations on systematic approaches for assessing and intervening on occupational factors [14]. Moreover, OH guidelines seldom describe implementation strategies [6], which are aimed at assisting users with the implementation of recommendations [15].

One way to improve validity, appropriateness, applicability and ultimately usage of guidelines is by encouraging participation of end-users in the process of guideline development through communities of practice (CoP) [16]. A CoP is a group of people with a common interest who collaborate over an extended period to share ideas, solve problems, and create knowledge [17]. CoP can play an important role in the development of practice guidelines as they capture and diffuse existing knowledge to help people improve their practice by providing a forum to identify solutions to common problems and a process to collect and evaluate best practices [17]. By creating and sharing knowledge that has meaning for practitioners the uptake of best practice will likely increase. CoPs have previously been used in the development of a primary care low-back pain guideline [16].

A common problem observed with practice guidelines is that they often suffer from shortcomings in the guideline development process, including a lack of transparency of the development groups’ methodologies, failure to convene multi-disciplinary guideline development groups and overall failure to use rigorous methodologies in the development [18]. This paper contributes to filling a current gap in the literature in this field, by providing a detailed description of the development of an OH practice guideline aimed at the management of non-specific LBP using a multidisciplinary CoP approach.

## Methods

The first aim of this study is to describe the development process, which will establish transparency with regards to the methodologies used to develop the guideline. The second aim is to describe the evaluation of the development process. This will provide valuable insights regarding the feasibility of the development process, especially regarding the involvement of practitioners through a CoP and will have important implications for the development of future guidelines in this and other settings.

### Development of the OH practice guideline

The guideline was developed based on recommendations of existing manuals on guideline development, including the manual of the National Institute for Health and Clinical Excellence (NICE). The process consisted of four phases: preparing for guideline development, systematically reviewing the evidence, drafting the guideline and reviewing the guideline [19].

#### Phase 1: preparing for guideline development

##### Composition of the community of practice

The development process started in 2012 by forming the CoP. Criteria on the composition and size of the group were set by the project team, which consisted of three researchers in the field of occupational health, implementation and LBP. The first criterion was that the CoP should be multidisciplinary and balanced, comprising of at least two representatives each of OH occupations (occupational physicians, occupational nurses, ergonomists/physical therapists, health and safety engineers, health educators and psychologists) involved with the management of non-specific LBP. The second criterion was that the CoP should be relatively small (max 15 members) to enable productive group-discussions and group work. The final criteria stated that the group should be a balance between OH professionals from in-house OHS units and from private OHS providers, small and large OHS and different geographical regions within Sweden. Group-members were invited as representatives of their field or discipline. The project-team was also part of the CoP. One member of the project-team was group chair and responsible for all meetings. Disclosure of interest was discussed with the group-members. It was underscored that the recommendations should solely be based on evidence applicable in OHS settings and not on specific methods or processes used within OHS units. None of the group-members or researchers had a conflict of interest.

##### External advisors and reviewers

During the development process an external advisory group assisted the project-team with giving presentations during the meetings, providing advice and literature suggestions. The project-team decided which experts should be included. The following inclusion criteria were stated: mastery of the clinical topic, expertise on relevant research and studies in progress, and/or practical experience. The advisory group (*n* = 7) contained an orthopedic specialist, a researcher in work-related musculoskeletal disorders, a researcher specialized in occupational and environmental medicine and the following practitioners working within OHS: an occupational physician, a psychologist and two ergonomists/physical therapists.

A reference group of external reviewers was responsible for reviewing the developed guideline. The reference group (*n* = 12) was selected by the project-team, a decision was made that the group should consist of both researchers and OH practitioners. The group consisted of high profile researchers in the field of LBP (*n* = 6), occupational physicians (*n* = 2), ergonomist/physical therapist (*n* = 1), psychologist (*n* = 1), and representatives of the Occupational Health Physician Society (*n* = 1) and the Swedish Association for Occupational Safety and Health (*n* = 1). All communication with external reviewers was conducted by email; all reviewers gave permission to be listed as external reviewers in the guideline.

##### Planning and set up of the meetings

In total 11 meetings were organized by the project-team (June 2012–December 2013). All meeting were held at the national organization of OHS in Stockholm, Sweden. Table 1 presents the structure and content of the meetings. The meetings included presentations of experts in the field, discussions, group-work and home-assignments. During the meetings group members had intermediate email contact. The minutes, PowerPoint presentations and reading/working material were posted on closed pages of the branch organization’s website, which group members had access to. During the first two meetings clear group roles/norms were discussed, including having respect for each other, not being judgmental, being inclusive and listening to each other. Moreover, group members were asked to share their expectations of their participation.

**Table 1:** Steps and content of the meetings of the community of practice

Phases	Meetings	Content
Phase 1: Preparing for guideline development 1.1. Selecting the topic	Meeting 1 (2012-06-13)	Introduction round
		PowerPoint presentation by group chair on the goal of the project and program, and on evidence-based practice
		Pairwise and group-discussion on OHS tasks and evidence-based practice within OHS
		Discuss group aims, norms of behavior and practicalities
	Task for next meeting	Describe expectations and prerequisites of participation
	Meeting 2 (2012-09-11)	Implementation expert gives PowerPoint presentation on implementation research, including concepts and theories
		Present a summary of expectations and prerequisites of participants
		Pairwise and group-discussion on topic selection for the guideline
	Task for next meeting	Make an inventory of methods used within the own OHS, discuss what is needed within the OHS
Phase 1: Preparing for guideline development 1.2. Determining the scope	Meeting 3 (2012-12-12)	PowerPoint presentation of back pain and diagnostics by external orthopedic specialist
		PowerPoint presentation on evidence-based assessment and treatment of LBP, with a focus on psychosocial factors
		Group discussion on methods used within the OHS and on OHS protocol of working with LBP
		Formulate objectives and target-group of guideline
	Task for next meeting	Make an inventory of methods used within the own OHS with regards to diagnostics and treatment of LBP
Phase 2: systematically reviewing the evidence 2.1 Establishing clinical questions	Meeting 4 (2013-02-13)	PowerPoint presentation regarding the scientific evidence for assessment tools and interventions related to the work environment
		PowerPoint presentation of an example of the Dutch back pain guideline of the Netherlands Society of Occupational Medicine
		Small and big group discussions on content and format (flow-chart) of the guideline and on implementation, including barriers, facilitators, and strategies
	Task for next meeting	Describe current protocol of LBP assessment and treatment within own OHS regarding who does what – in order to identify target users. Startup internal working-group within own OHS
Phase 2: systematically reviewing the evidence 2.2. Appraising research	Meeting 5 (2013-03-27)	PowerPoint presentation on valid methods for ergonomic assessment by a professor in work-related musculoskeletal disorder research
		Each participant presents current practice with regards to LBP assessment and treatment
		Group discussion flow-chart for treatment of LBP
	Task for next meeting	Work on flow-chart – screening-questions
	Meeting 6 (2013-06-19)	A Swedish example of an OHS work with the treatment of LBP
		Ergonomics present ergonomic assessment tool proposal appropriate for OHS setting
		Group discussions on sent out material
Phase 3: Drafting the guideline	Meeting 7 (2013-08-16)	PowerPoint presentation regarding implementation strategies by implementation expert
		PowerPoint presentation of NICE-guideline for the early management of non-specific LBP.
		Interdisciplinary group-work regarding ergonomist observation methods and interventions, and behavior change methods and interventions
	Task for next meeting	Individual groups work on drafting text
	Meeting 8 (2013-09-20)	Individual groups continue with drafting text for the guideline
		Group discussion on progress and challenges with drafting text
	Meeting 9 (2013-10-15)	PowerPoint presentation on KOF, a dialog-method to assess work capacity and work demand, by external expert
		Individual groups continue with drafting text for the guideline
Reviewing the guideline	Meeting 10 (2013-11-12)	Adapt text to internal and external review comments
	Meeting 11 (2013-12-16)	

*Note. OHS* occupational health service, *LBP* low back pain

##### Selecting the guideline topic

The aim of the first two meetings was to select and prioritize a guideline topic. This was based on discussions on (the lack of) evidence-based practice within the OHS, the relevance of the topic (i.e. how common the health problem is in OHS), areas where there is a wide variation in practice, the perceived need for a guideline in a specific topic and areas where there is sufficient good quality evidence. To facilitate discussions a presentation was given on evidence-based practice, including definition and concepts. At this stage a choice was made to develop an OH guideline for the management of non-specific LBP.

##### Determining the scope of the guideline

During meeting 3 the aim of the guideline, the target users and the target population were discussed. The CoP decided that the guideline should provide guidance on: 1) methods to identify those with non-specific LBP; 2) valid assessment tools to identify physical and psychosocial risk factors for non-specific LBP; 3) effective interventions supported by current best evidence for the management of non-specific LBP and 4) implementation of the recommendations. It was underscored that the guideline should be easy to use, build on the OHS’s multidisciplinary competence and their expertise in workplace and workers’ health. A decision was made that the target users should be those OHS professionals who are involved in supporting employees with non-specific LBP. As this often varies among practitioners within the OHS the CoP agreed that the guideline should not specify which specific occupations should be involved in the different steps in the process. The target population includes individuals with non-specific LBP and excludes individuals with specific LBP. The CoP agreed that those individuals with specific LBP should be referred to other medical specialists outside of the OHS.

#### Phase 2: systematically reviewing the evidence

##### Establishing clinical questions

From meeting 4–9, presentations were given by (external) experts on topics related to answering the following identified clinical questions: 1) which method should be used to identify those with non-specific LBP; 2) what assessment tools or processes should be used to identify risk factors for non-specific LBP; 3) what are the most effective interventions for non-specific LBP and 4) what is needed to implement the recommendations within the OHS? These questions were defined on the basis of discussions on current practice within the OHS when managing non-specific LBP.

##### Literature search

The literature search for evidence to answer the clinical questions was conducted by the project-team in consultation with the external advisory group. It was also guided by discussions held within the CoP and practical experiences. The starting point of the search was a Swedish governmental report, which provides recommendations for interventions aimed at non-specific LBP [20]. The recommendations in the report are based on seven existing international guidelines (e.g. European guidelines for the management of chronic nonspecific low back pain (2006) [21], Alberta Clinical Practice Program’s guideline for the management of LBP [22] and the National Institute for Health and Clinical Excellence’s guideline for the early management of persistent non-specific LBP (2009) [23], and systematic reviews [24–26]). These reports are mainly aimed at managing non-specific LBP within the primary care setting. Complimentary searches were conducted by the project-team for relevant articles related to diagnosis, assessment and intervention for non-specific LBP within a worksite/OH setting and for articles published after the reports. A systematic search was conducted through PubMed, the Cochrane Database of Systematic Reviews (1966 through 2013), reference lists and grey-literature.

##### Home-assignments

During the guideline development process CoP-members received several home-assignments, which included listing assessment tools and questionnaires used in their OHS for the management of non-specific LBP, reading proposed literature and writing draft texts for the guideline. Moreover, CoP-members were encouraged to set up internal groups at their OHS with colleagues involved in the management of non-specific LBP. The aim of these “local” groups was to receive input from additional OH professionals, in order to enhance the implementability of the guideline.

#### Phase 3: drafting the guideline

The drafting of the guideline was conducted by the CoP between meetings 7–10. The group was divided into smaller groups of similar occupations (e.g. occupational physician) led by a researcher, each group was responsible for drafting a part of the guideline. The researchers guided and supported the smaller groups in systematically and critically appraising available evidence/literature and in drafting text for the guideline. During the meetings each group presented their findings and during following discussions, consensus agreements on what to include in the guidelines were obtained. These agreements were based on the strength of the evidence in combination with applicability within the OHS setting. The recommendations are based on identified evidence (e.g. [24, 27–30]), discussions and practical experiences. The guideline is divided into six sections: introduction, flow-chart, assessment, intervention, implementation and appendices. The assessment, intervention and implementation sections contain descriptions of the recommendations, scientific evidence for the recommendations, and examples of validated tools. The appendices included questionnaires, scoring protocols, a checklist for implementing the guideline into practice, work-material for planning interventions and examples of an invitation-letter to employers and an information-sheet on how LBP influences everyday life.

#### Phase 4: reviewing the guideline

Between the 10-11th meeting the draft of the guideline was reviewed both within the CoP, but also externally by the reference group. The guideline was checked for accuracy, comprehensiveness and balance of the scientific evidence and validity of the rational for recommendations [31]. Moreover feedback was given on the clarity and feasibility of the recommendations. Adaptations were made to the guideline in accordance with reviewers’ comments. The project-team discussed the rationale for modifying or not modifying the guideline in response to reviewers’ comments. A journalist specialized in occupational health and safety information and a graphic designer were assigned for lay-out and text editing.

In January 2014 the guideline was launched during a national seminar. During the seminar CoP members were involved in presenting the guideline to OH professionals and employers. Information on the guideline is also provided by short webinars.

### Evaluating the guideline development process

Following the recommendations of Linnan and Steckler [32] five process outcomes were assessed, namely recruitment, reach, context, satisfaction and fidelity. In addition a measure of feasibility was assessed [33] (Table 2). Context, satisfaction and feasibility were assessed on the OH professional level by asking the OH professionals within the CoP (*n* = 13) to complete a process-questionnaire at 12 months after the start of the development process. Fidelity was measured at the level of the project-team. Finally, during the first meeting group-members wrote down their expectations of participating in the CoP, which were summarized and followed up by discussing goal fulfilment during the last meeting. The researcher responsible for the process-evaluation was experienced in implementation research and was not involved in the guideline development process. The method of assessment of all process outcomes is described in more detail below.

**Table 2:** Description and methods of measurement for the process evaluation

Process outcome	Description	Method of measurement
Recruitment	Procedures used to approach and attract CoP members	Administrative data, emails
Reach	The proportion of the CoP members that participates in the meetings	Attendance form, emails
Context	Aspects of the larger social, political, and economic environment that can influence the development process	Observation questionnaire
Satisfaction	The perception of the CoP members that the development process is satisfactory and agreeable	Questionnaire
Feasibility	The extent to which the development process can be successfully used or carried out in this particular setting	Questionnaire
Fidelity	The extent to which the CoP meetings are delivered as planned by the project-team.	Observation, minutes of meetings

*Note*. Adapted from Linnan et al. and Proctor and et al. [32]

#### Recruitment

Recruitment refers to the procedures used to approach and recruit OH professionals to participate in the CoP [32]. In order to meet the selection criteria a purposive sampling approach was applied. The methods that were used to recruit participants, the number of individuals potentially interested, and the reasons for not participating were documented.

#### Reach

Reach is defined as the extent to which the intervention contacts are received by the target population [32]. In this study reach was operationalized as the number of CoP members that attended the meetings. A member of the project-team completed attendance forms during every meeting. The average attendance was calculated. Reasons for discontinuation were registered.

#### Context

Context refers to those factors within the larger social, political, and economic environment that may influence the implementation of an intervention [32]. Contextual factors were assessed that might influence the CoP members’ participation in the guideline development process. These factors were assessed by observation and questionnaire. The process evaluator was present during all meetings and registered any possible contextual factors that might influence participation in the group. In the questionnaire CoP members indicated on a five-point Likert scale (no agreement- full agreement) whether they agreed with the following four statements: my involvement in the guideline development group is supported by my boss; my involvement within the guideline development group is supported by my colleagues; I have received good economical prerequisites (e.g. reimbursement of travel costs) for participating in the guideline development group; I have had the possibility to work on the guideline development in between meetings during working hours. Results are presented by merging alternative five (full agreement) and four on the point Likert scale as “agreement”.

#### Feasibility

Feasibility is defined as the extent to which the development process could be successfully used or carried out within a particular setting or a certain population [33]. Three statements assessed the feasibility of whether the guideline development process could be successfully carried out within this setting. In the questionnaire CoP members indicated on a five-point Likert scale (no agreement- full agreement) whether they agreed with the following three statements: it is possible for me to combine the guideline development group with my work at the OHS; the time-scheduling (i.e. frequency, duration) of the meetings has worked well; the guideline development group has a good working method for the development of guidelines. Results are presented by merging alternative five (full agreement) and four on the point Likert scale as “agreement”.

#### Fidelity

Fidelity is defined as the extent to which the implementation of an intervention adheres to the protocol or program model originally developed. It represents the quality and integrity of the intervention as conceived by the developers. Fidelity is a function of the intervention providers [32]. Fidelity was assessed by the project-team who examined whether the frequency and duration of the meetings was as planned, whether presentations by external experts were given as planned and whether CoP members were engaged (e.g. participated in discussions, completed home-assignments) in the development process as anticipated.

#### Satisfaction

Satisfaction refers to the perception of the CoP-members that the development process was satisfactory or agreeable. In the questionnaire CoP-members indicated on a five-point Likert scale (no agreement- full agreement) whether they agreed with the eleven statements related to the content of the meetings (e.g. presentations that have been held during the meetings have been interesting) and the guideline development process (e.g. working within the group has lived up to my expectations). Results are presented by merging alternative five (full agreement) and four on the point Likert scale as “agreement”.

### Ethical considerations

According to Swedish law governing ethical review of research involving humans, this study did not require ethical approval. All group-members were informed during the first meeting that the guideline development process would be evaluated, group members were informed about the purpose of the evaluation and reassured about confidentiality. No sensitive data was collected and data are presented so that individual participants remain anonymous.

## Results

### Evaluation of the guideline development process

#### Recruitment

Occupational physicians, occupational nurses, ergonomists/physical therapists, behavioral scientists, health educators, psychologists and occupational health and safety engineers were recruited in 2012 by open invitation send out to OHS by email, and published on the project team’s website and blog. The recruitment was based on purposive sampling in which a selected group of 130 OH professionals with the identified occupations were sent an email describing the plans to set up a CoP responsible for the development of an OH practice guideline. The email also described practicalities, including what it would entail to become a member and details regarding the frequency and duration of the meetings. Interested individuals were instructed to send a description of themselves and their interest in participation, to the project-team. Twenty-six individuals (20%) expressed an interest in participating. Based on the set inclusion criteria the project-team accordingly purposively selected thirteen OH professionals representing a diversity of clinical backgrounds, geographical regions, in-house and private OHS, and small and large OHS. The characteristics of the group members are described in Table 3.

**Table 3:** Baseline characteristics of the OH professionals within CoP (*n* = 13)

Characteristics		SD
Age (mean, years)	49.6	8.4
Female (%)	76.9	
Work-related characteristics		
Years working experience (mean)	26.2	9.6
Years working within the OHS (mean)	12.2 (7.8)	7.8
Employment within OHS (% full-time)	76.9%	
Job title (n)		
Occupational physician	3	
Ergonomist/physical therapists	2	
Occupational nurse	2	
Health and safety engineer	2	
Health educator	2	
Psychologist	2	

*Note. SD* standard deviation, *OHS* occupational health service

#### Reach

Group members attended on average 7.5 out of 11 meetings (~68%). After five months one member discontinued participation due to maternity leave and was replaced. Three additional members discontinued due to dissatisfaction with the group-process (*n* = 1) and change of job (*n* = 2), but were not replaced as this occurred during a later stage in the development process. Twelve months after the start of the development process the CoP consisted of ten OH professionals.

#### Expectations of participation

CoP-members described 19 different expectations, mostly related to evidence-based methods; to gain knowledge about evidence-based methods and how to implement these methods into practice. Group-process expectations were also identified, including equal sharing of experiences, respect for each other’s integrity and for other professions’ traditions and ways of working and the importance of impartibility. Some group-members indicated research related expectations, such as developing a platform for research-projects, contributing to practice-based research and formulating proposals for research-projects. Finally, some group-members expected that the OH practice guideline will be used as a tool to ensure quality of practice, as a marketing strategy for evidence-based practice and as a procurement requirement specification.

#### Context

The response rate to the process questionnaire was 80% (*n* = 8). Of the eight CoP members who completed the questionnaire seven indicated that they had received good economical prerequisites for their involvement in the group. Half of the respondents agreed with the statements that they had received support from colleagues and management for their involvement within the group. Three respondents (37.5%) indicated that they had received good prerequisites regarding being able to work on the guideline development between meetings (Table 4).

**Table 4:** OH professionals’ within CoP (*n* = 8) agreement with statements on context, satisfaction and feasibility of the process

Statements	Agreement n (%)
Context	
My involvement within the guideline development group is supported by my colleagues	4 (57%)
My involvement within the guideline development group is supported by my boss	4 (50%)
Have you received good economical prerequisites for participating in the guideline development group	7 (87.5%)
Have you received good prerequisites regarding time to be able to actively participate in the guideline development between meetings	3 (37.5%)
Satisfaction	
The task of the guideline development group is clear and understandable	5 (62.5%)
The presentations given during the meetings have been interesting	8 (100%)
The presentations are a necessary component of the guideline development process	8 (100%)
Overall I am satisfied with the meetings we have had	5 (62.5%)
A multi-professional set-up of the guideline development group is necessary for the development of the guideline	7 (87.5%)
By participating in the guideline development group my knowledge of evidence-based methods has increased	8 (100%)
The work done within the guideline development group has led to the development of the guideline	8 (100%)
I feel that I am an active member of the group	8 (100%)
I am satisfied with the topic (management of LBP) of the guideline	4 (57%)
Being part of the guideline development group has been valuable for developing practices within the OHS	8 (100%)
The work done within the guideline development group has lived up to my expectations	4 (50%)
Feasibility	
The guideline development group has a good working method for the development of guidelines?	6 (75%)
The time-scheduling (i.e. frequency, duration) of the meetings has worked well	1 (12.5%)
It is possible for me to combine the task of the guideline development group with my work at the OHS?	7 (87.5%)

*Note. LBP* low back pain, *OHS* occupational health services

Observations of the group process revealed that the first few meetings were mainly characterized by developing good interpersonal relations and trust between the group members. Moreover, during the entire guideline development process there was an uneven distribution of input among participants, some group members participated more heavily in the discussions and others in the drafting of the guideline. Finally, the observations showed that the researchers within the group took on an active role in leading and supporting the process.

#### Feasibility

Feasibility was assessed with three statements. 87.5% agreed with the statement that it is possible to combine the task of the guideline development group with my work at the OHS, moreover 75% agreed that the group had a good working method for the development of the guidelines. However, only one CoP member agreed that the time-scheduling worked well.

#### Satisfaction

All group members agreed with six of the twelve statements on satisfaction. They agreed that the work done within the group led to the development of the guideline, that they were an active member in the group, that their knowledge of evidence-based methods had increased and that being part of the group has been valuable for developing practices within the OHS. Moreover, they agreed that the presentations given by the advisory/reference group were interesting and a necessary part of the guideline development process. 87.5% agreed that the multi-professional set-up of the group was necessary for the development of the guideline. 62.5% agreed that the task of the guideline development group was clear and understandable and that they were overall satisfied with the meetings they had. Half of the respondents indicated that the work done within the group had lived up to their expectations and that they were satisfied with the topic of the guideline.

#### Fidelity

Observations showed that the guideline development process was on two fronts not developed as intended. Firstly, it was planned that the choice of guideline topic would be based on a perceived need of the OH professionals within the group. However, due to a lack of input regarding a specific need, the choice was based on the researchers’ knowledge regarding available evidence in the area of non-specific LBP and the wide variation in current praxis. Secondly, the time-scheduling of the meetings was adapted during the development process. Meetings were planned more frequently and longer, as the OH professionals within the group indicated that it was difficult to schedule time to work on the guideline development between meetings.

## Discussion

This paper describes the development of an OH practice guideline for the management of non-specific LBP by including the end-users in the process and includes a process evaluation of the development process. It provides valuable information on the feasibility of including end-users in the development process, an important prerequisite to improve the use of guideline and facilitate evidence-based practice. There is to date a demand for transparency in the development of practice guidelines, this in order to ensure that guidelines are of high quality, trustworthy and implementable [18] and to reduce any potential biases. With the paper’s detailed description of the rigorous methodologies used by the multidisciplinary CoP in the development of the OH practice guideline we contribute to the dearth of publications in this field. The developed guideline is closely in line with the US Institute of Medicine’s standards for developing rigorous, trustworthy clinical practice guidelines [34]; it is based on available evidence, developed by a knowledgeable, multidisciplinary group of experts and representatives from the OHS, based on an explicit and transparent process that minimizes biases and includes a description of the relationship between the suggested interventions and health outcomes.

This paper also includes a thorough process evaluation. Overall the process evaluation showed that respondents were satisfied with the content and necessity of the presentations given during the meetings, that participation in the CoP increased their knowledge of evidence-based practice and that it has value for developing practice within the OHS. With regards to feasibility, respondents indicated that the CoP had good working methods for the development of the guideline and that is was possible to combine the tasks of the CoP with their work at the OHS. However, the process evaluation also indicated several challenges with the development process.

A first challenge concerns the recruitment: 20% of those contacted showed an interest in participating. This has likely resulted in a CoP representing a selective and motivated group of OH professionals. One reason for this relatively low interest is that the guideline was the first OH practice guideline to be developed by applying a CoP-approach, possibly resulting in uncertainty among OHS regarding expectations and demands of the development process. The recruitment was successful in selecting a balanced multidisciplinary CoP representing the different occupations, types of OHS and geographical locations.

The second challenge is the level of participation, a common problem in studies using CoPs [35]. The number of members as well as the participation level between members varied over time. As indicated by Wenger et al. (2002) it is unrealistic to expect that all members participate equally, as individuals have different reasons for participating in a CoP [36]. The participation level could have been influenced by the contextual factors, such as lack of support from management and colleagues, and lack of good prerequisites regarding time to be able to actively participate in the development process between meetings. An additional factor that could have influenced the participation level is the time-scheduling, which was not deemed as feasible by 87.5% of respondents. The lack of management support raises an interesting question concerning the employer’s incentive for letting their employee participate in the CoP. The participation level could also have been influenced by the fact that not all group members were satisfied with the meetings, the clarity of the tasks and the topic chosen.

A third challenge relates to group dynamics. Observations showed that the first few meetings were characterized by little sharing and exchanging of experiences between the OHS professionals. A successful CoP is characterized by mutual engagement and exchange that involves sharing, interacting and supporting each other [37]. Factors that have been proposed to influence successful collaboration within CoPs are trust building, perceived value in information sharing and willingness to engage [38]. In Sweden, many OHS exist within a private context, and are business competitors. Therefore it is likely that during the first meetings feelings of trust had to be built before members were willing to share experiences with each other. Several of the members expectations (e.g. “all should be willing to contribute to the work and share their experiences” and “group members should be impartial having the employee’s benefit in focus and not that of their own employer), support this assumption.

A final challenge concerns trust building between the OHS professionals and researchers, which also influenced the group process during the first few meetings. This was most likely a result of the different expectations of the OHS professionals and the researchers regarding the applied bottom-up approach. As suggested by Bindels et al. a decision was made by the researchers to use a bottom-up approach [35] and involve group members in the selection of the guideline topic. However, as the process-evaluation showed this was not applied as intended, instead a more top-down approach was applied, in which the researchers selected the guideline topic. During discussions it became apparent that the OHS professionals expected that the researchers would take on a more leading role in the development process. The process evaluation showed that only half of the respondents of the questionnaire indicated that the work done within the group had lived up to their expectations and half indicated that they were satisfied with the topic of the guideline. The CoP members valued the presentations by the advisory/reference group as valuable; this may also be an expression of the expectation of getting scientific knowledge presented, rather than actively searching for it.

### Strengths and limitations

Strengths of the present study include the structured approach of guideline development by using a multidisciplinary group reflecting the Swedish OHS context, increasing the applicability of the guideline. A first limitation relates to the disclosure of interest procedure of the CoP, no standardized forms for disclosure of interests were used. However, during the development process the importance of being open minded to new forms of practice, including assessment instruments and interventions that group members are unfamiliar to, was repeatedly discussed. In the current guideline development process a standardized form for disclosure of interest is included in the process. The second limitation is the choice of guideline topic, which was not implemented as intended. A risk of having the researchers in the CoP identify the topic is that the it may not reflect a current need in practice for a guideline on this topic. In the ongoing guideline development process, a topic prioritization group consisting of representatives from employers and employee organizations, similar to those within the US Preventive Service Task Force (https://www.uspreventiveservicestaskforce.org/), is responsible for prioritizing the guideline topic. In the invitation to the OHS professionals they now apply for a CoP with a predefined topic such as mental ill health. A third limitation relates to the process-questionnaire used; the questionnaire items were not validated. However, the items were based on existing items used in previous studies (e.g. [39]) and on input from an expert-group in the field of process evaluation. A fourth limitation relates to the data collected on feasibility, no data were collected on time spent on the development process in-between meetings or on the cost made during the process. We were therefore unable to weigh costs against benefits; this information would be valuable for further use of the approach. A final limitation concerns the response rate to the questionnaire; two CoP-members did not complete the questionnaire; this could indicate a dissatisfaction with the development process.

## Conclusion

The described development process is a feasible process for guideline development. This paper has important implications for future guideline development; it provides valuable information on how practitioners can be included in the development process, with the aim of increasing the implementability of the developed guidelines. In order to enhance the field of guideline development it is imperative that end-users are included in the development and that approaches to include end-users are evaluated and described. It was not in the scope of the study to present the content of the guideline, neither to assess the usage of the guideline within the OHS. Future studies will be conducted on guideline usage, including barriers and facilitators of implementation and implementation strategies that could further enhance uptake of the guideline.

## Abbreviations

CoP: Community of practice
LBP: Non-specific low back pain
OH: Occupational health
OHS: Occupational health services
